# Evolução e Estado Atual das Práticas de Implante Transcateter de Válvula Aórtica na América Latina – Estudo WRITTEN LATAM

**DOI:** 10.36660/abc.20210327

**Published:** 2022-06-06

**Authors:** Fernando Luiz de Melo Bernardi, Henrique Barbosa Ribeiro, Luis Nombela-Franco, Enrico Cerrato, Gabriel Maluenda, Tamim Nazif, Pedro Alves Lemos, Matias Sztejfman, Pablo Lamelas, Dario Echeverri, Marcelo Antonio Cartaxo Queiroga Lopes, Fábio Sândoli de Brito, Alexandre A. Abizaid, José A. Mangione, Helene Eltchaninoff, Lars Søndergaard, Josep Rodes-Cabau

**Affiliations:** 1 Universidade de São Paulo Faculdade de Medicina Hospital das Clínicas Instituto do Coração São Paulo SP Brasil Universidade de São Paulo – Faculdade de Medicina Hospital das Clínicas – Instituto do Coração, São Paulo, SP – Brasil; 2 Hospital Samaritano Paulista São Paulo SP Brasil Hospital Samaritano Paulista, São Paulo, SP – Brasil; 3 Hospital Sírio-Libanês Hemodinâmica e Cardiologia Intervencionista São Paulo SP Brasil Hospital Sírio-Libanês – Hemodinâmica e Cardiologia Intervencionista, São Paulo, SP – Brasil; 4 Hospital Clínico San Carlos Madrid Espanha Hospital Clínico San Carlos, Madrid – Espanha; 5 San Luigi Gonzaga University Hospital Orbassano Itália San Luigi Gonzaga University Hospital, Orbassano – Itália; 6 Hospital Clínico San Borja Arriarán Santiago Chile Hospital Clínico San Borja Arriarán, Santiago – Chile; 7 Columbia University Medical Center New York EUA Columbia University Medical Center, New York – EUA; 8 Hospital Israelita Albert Einstein São Paulo SP Brasil Hospital Israelita Albert Einstein, São Paulo, SP – Brasil; 9 Sanatorio Finochietto Buenos Aires Argentina Sanatorio Finochietto, Buenos Aires – Argentina; 10 Instituto Cardiovascular de Buenos Aires Buenos Aires Argentina Instituto Cardiovascular de Buenos Aires, Buenos Aires – Argentina; 11 Fundacion Cardioinfantil Instituto de Cardiologia Bogota Colômbia Fundacion Cardioinfantil Instituto de Cardiologia, Bogota, Cundinamarca – Colômbia; 12 Hospital Alberto Urquiza Wanderley João Pessoa PB Brasil Hospital Alberto Urquiza Wanderley, Cabedelo, João Pessoa, PB – Brasil; 13 Beneficência Portuguesa de São Paulo São Paulo SP Brasil Beneficência Portuguesa de São Paulo, São Paulo, SP – Brasil; 14 Hôpital Charles Nicolle Rouen Normandy França Hôpital Charles Nicolle, Rouen, Normandy – França; 15 Copenhagen University Hospital Kobenhavn Dinamarca Copenhagen University Hospital, Kobenhavn – Dinamarca; 16 Institut Universitaire de Cardiologie Et de Pneumologie de Québec Quebec Canadá Institut Universitaire de Cardiologie Et de Pneumologie de Québec, Quebec – Canadá

**Keywords:** Substituição da Valva Aórtica Transcateter, Estenose da Valva Aórtica, América Latina

## Abstract

**Fundamento:**

Implante transcateter de valva aórtica (TAVI) é um procedimento adotado em todo o mundo e suas práticas evoluem rapidamente. Variações regionais e temporais são esperadas.

**Objetivo:**

Comparar a prática de TAVI na América Latina com aquela no resto do mundo e avaliar suas mudanças na América Latina de 2015 a 2020.

**Método:**

A pesquisa foi realizada em centros de TAVI em todo o mundo entre março e setembro de 2015, e novamente nos centros latino-americanos entre julho de 2019 e janeiro de 2020. As seguintes questões foram abordadas: i) informação geral sobre os centros; ii) avaliação pré-TAVI; iii) técnicas do procedimento; iv) conduta pós-TAVI; v) seguimento. As respostas da pesquisa dos centros latino-americanos em 2015 (LATAM15) foram comparadas àquelas dos centros no resto do mundo (WORLD15) e ainda àquelas da pesquisa dos centros latino-americanos de 2020 (LATAM20). Adotou-se o nível de significância de 5% na análise estatística.

**Resultados:**

250 centros participaram da pesquisa em 2015 (LATAM15=29; WORLD15=221) e 46 na avaliação LATAM20. No total, foram 73.707 procedimentos, sendo que os centros WORLD15 realizaram, em média, 6 e 3 vezes mais procedimentos do que os centros LATAM15 e LATAM20, respectivamente. Os centros latino-americanos realizaram menor número de TAVI minimalista do que os do restante do mundo, mas aumentaram significativamente os procedimentos menos invasivos após 5 anos. Quanto à assistência pós-procedimento, observaram-se menor tempo de telemetria e de manutenção do marca-passo temporário, além de menor uso de terapia dupla antiplaquetária nos centros LATAM20.

**Conclusão:**

A despeito do volume de procedimentos ainda significativamente menor, muitos aspectos da prática de TAVI nos centros latino-americanos evoluíram recentemente, acompanhando a tendência dos centros dos países desenvolvidos.

## Introdução

O implante transcateter de válvula aórtica (TAVI) vem sendo adotado no mundo todo para o tratamento da estenose aórtica importante sintomática em pacientes de vários perfis de risco. Este feito foi alcançado ao longo de mais de uma década de avanços da tecnologia e da assistência ao paciente. Como consequência, as práticas de TAVI têm evoluído rapidamente, resultando em melhora significativa dos desfechos clínicos.^1–4^

Na América Latina, os primeiros procedimentos de TAVI foram realizados em 2008 no Brasil e na Colômbia.^5,6^ A despeito do crescimento regular dos casos observado desde então, existe a preocupação quanto à adoção das práticas mais atuais pelos centros da América Latina.^8–10^ Nos países em desenvolvimento, disparidades na prática de procedimentos médicos de alto custo podem ser exacerbadas devido a vários fatores, como sistemas de saúde de menor renda, menores volumes de procedimento nos centros, menor experiência dos operadores e indisponibilidade de certos dispositivos. Compreender tais diferenças é crucial para o melhor entendimento das práticas contemporâneas e para a busca de padronização dos serviços. Além disso, pode auxiliar no desenvolvimento de políticas pelos reguladores locais para a maior difusão da TAVI nas populações desassistidas, considerando que publicações científicas latino americanas sobre o tema são limitadas.

Portanto, os objetivos geral e secundário deste estudo foram: i) comparar a prática da TAVI entre centros latino-americanos e do resto do mundo com base em dados obtidos na pesquisa WRITTEN de 2015; ii) avaliar as alterações na prática de TAVI na América Latina após 5 anos através de nova aplicação da pesquisa no continente.

## Métodos

**Figura 1:**
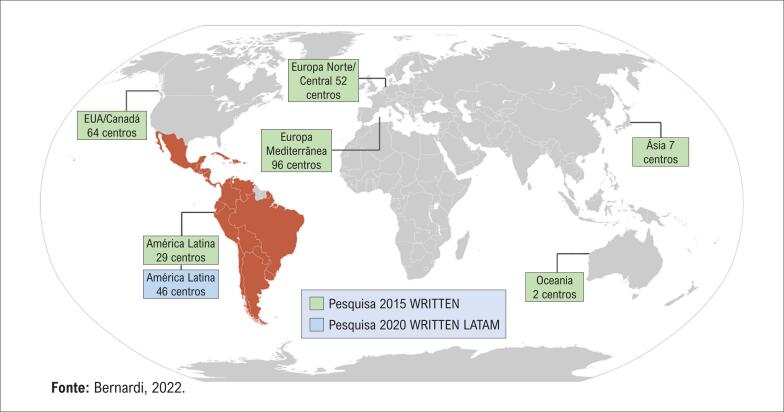
Distribuição geográfica dos centros participantes das pesquisas de 2015 e 2020.

A pesquisa WRITTEN 2015 foi um questionário com base na Internet, planejado para investigar as práticas nos centros de TAVI em todo o mundo. O desenho dessa pesquisa foi descrito anteriormente.^7^ Em resumo, pelo menos um expert de TAVI regional de cada país ou região foi contatado e convidado para distribuir a pesquisa WRITTEN 2015 localmente. A pesquisa foi promovida através de listas de mala direta de cardiologia intervencionista, anúncios de sociedades oficiais de cardiologia intervencionista, propaganda de *website* e e-mails personalizados para operadores de TAVI. Os convites foram distribuídos em diferentes áreas geográficas simultaneamente por 6 meses (de março a setembro de 2015). Uma segunda pesquisa foi realizada de julho de 2019 a janeiro de 2020, com método similar, envolvendo apenas centros da América Latina, sem um ponto de corte específico no número de procedimentos realizados por cada centro (Figura 1). A pesquisa consistiu em uma plataforma *online* hospedada em *website* de pesquisa colaborativa (www.cardiogrupo.org/TAVI/) com 59 questões abordando cinco domínios da TAVI (Tabela suplementar 1): (i) informação geral sobre o programa de cada instituição, (ii) seleção de pacientes, (iii) técnicas do procedimento e de imagem, (iv) conduta pós-procedimento e (v) seguimento. Foi solicitado que apenas um indivíduo de cada centro de TAVI respondesse à pesquisa e apenas um questionário por centro foi aceito.

### Análise estatística

Para a análise do estudo, as respostas correspondentes às práticas de TAVI dos centros latino-americanos em 2015 (LATAM15) foram usadas como referência. As variáveis categóricas foram expressas em frequências absolutas e porcentagens, sendo as contínuas expressas como mediana e intervalo interquartil (IIQ). Na comparação das variáveis categóricas, usou-se o teste exato de Fisher para avaliar a associação entre variáveis independentes (grupo dos centros) e dependentes (resultados do questionário) para respostas dicotômicas com valor de P bicaudal. Para as questões com mais de duas respostas possíveis, a associação entre variáveis independentes e dependentes foi testada com o teste do qui-quadrado. As variáveis contínuas foram comparadas usando o teste de Mann-Whitney, devido à sua distribuição não normal, confirmada pelo teste de Shapiro-Wilk, também com valor de P bicaudal. Adotou-se nível de significância de 5% para todas as análises estatísticas, que foram realizadas com o software GraphPad Prism, versão 7.0 (GraphPad Software, EUA).

## Resultados

**Figura 2:**
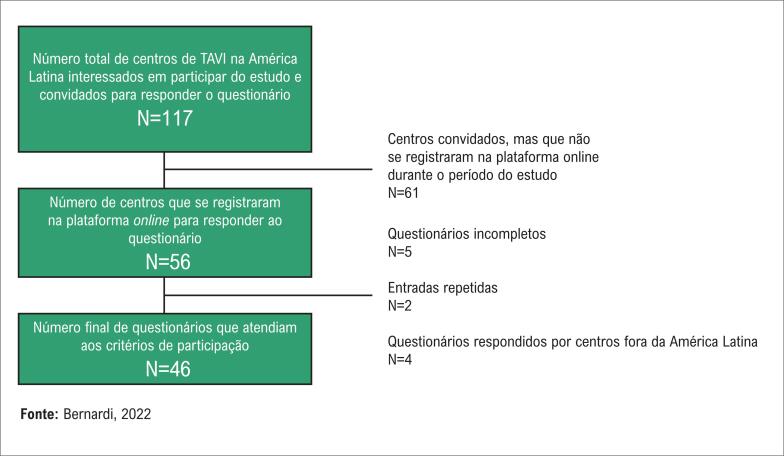
Fluxograma do arrolamento da pesquisa 2020 WRITTEN LATAM.

Como publicado anteriormente, 250 centros responderam ao questionário de forma adequada, sendo incluídos na pesquisa de 2015.^7^ Desses, 29 (11,6%) eram de centros latino-americanos (LATAM15). A Figura 1 ilustra a distribuição global dos centros. A Figura 2 resume a inclusão dos 46 centros participantes da pesquisa na América Latina em 2020 (LATAM20). Dos 296 questionários, 263 (88,8%) foram respondidos integralmente, enquanto nos demais, 80% das questões foram respondidas. Os poucos dados faltantes foram considerados completamente aleatórios e nenhum tratamento especial foi feito. Os nomes das cidades e países de todos os centros são listados nas Tabelas suplementares 2 e 3.

Ao término do estudo, o número total de TAVI realizadas pelos centros latino-americanos participantes em 2015 e 2020 (LATAM15 e LATAM20) e do resto do mundo (WORLD15) somaram juntos 73.707 procedimentos. Em comparação aos centros LATAM15, os centros WORD15 realizaram maior número de procedimentos no total de sua experiência (mediana: 34, IIQ: 12 a 101 vs. 200, IIQ: 84 a 453, p<0,001), assim como no ano anterior à finalização da pesquisa (mediana 12, IIQ: 5 a 23 vs. 60, IIQ: 27 a 110, p<0,001). Em comparação aos centros LATAM15, a experiência total dos centros LATAM20 foi cerca de duas vezes maior (mediana 62, IIQ: 22 a 138, p=0,08) mas apenas levemente superior à do ano anterior à finalização da pesquisa (mediana 16, IIQ: 6 a 30, p=0,29). Os resultados da pesquisa completa são apresentados nas Tabelas suplementares de 4 a 7.

### Avaliação pré-procedimento

**Figura 3:**
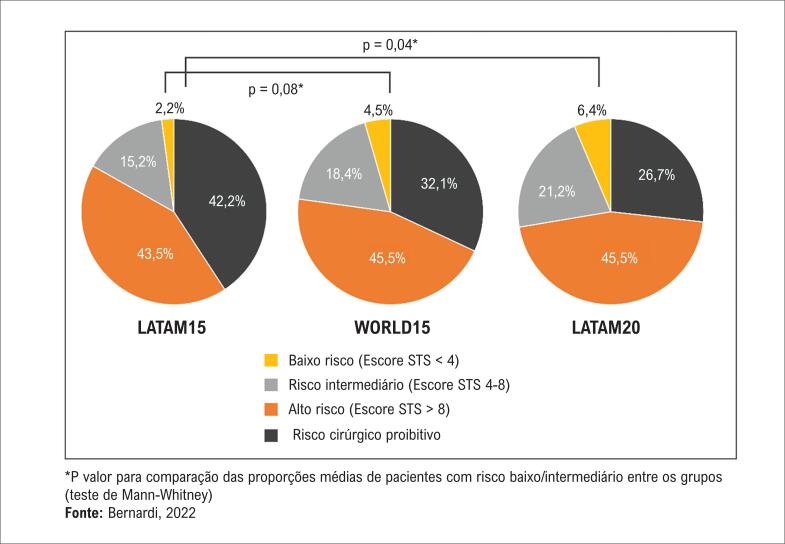
Proporções médias de pacientes tratados de acordo com o perfil de risco.

**Figura 4:**
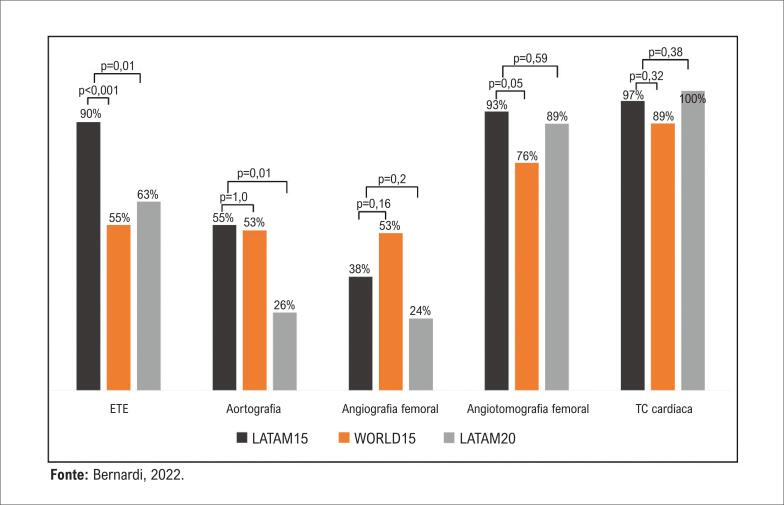
Comparação dos exames de imagem realizados rotineiramente antes do procedimento (% de centros). ETE: ecocardiograma transesofágico; TC: tomografia computadorizada.

Em todos os três grupos, a maioria dos pacientes submetidos a TAVI apresentava risco cirúrgico alto ou proibitivo. No entanto, ao se comparar os centros LATAM15 e LATAM20, observou-se aumento na proporção de pacientes com risco cirúrgico intermediário e baixo ao longo do tempo (Figura 3). Os centros WORLD15 apresentaram uma mediana maior de número de reuniões do ‘*heart team*’ mensais do que os centros LATAM15 (4, IIQ: 2 a 4 vs. 1, IIQ: 1 a 2, p=0,001), com leve aumento nos centros LATAM20 (1,5, IIQ: 1 a 4, p=0,27). O escore da Sociedade de Cirurgiões Torácicos Americana (STS) foi a ferramenta de estratificação de risco mais comum, usada rotineiramente por 90%, 69% e 98% dos centros LATAM15, WORDL15 e LATAM20, respectivamente. Por outro lado, apenas 28%, 47% e 39% dos centros, respectivamente, aplicaram testes de fragilidade de maneira rotineira. Com relação a testes de imagem pré-TAVI (Figura 4), praticamente todos os centros incluíram tomografia computadorizada cardíaca em sua prática. Ecocardiografia transesofágica antes do procedimento foi realizada de rotina mais frequentemente nos centros LATAM15.

Uma menor proporção de centros WORLD15 e LATAM20 administrou regularmente terapia de dupla antiagregação plaquetária (DAPT) antes de procedimentos transfemorais em comparação aos centros LATAM15 (45% e 56% vs. 83%, p<0,001 e p=0,02, respectivamente). Com relação ao tempo da intervenção coronariana percutânea (ICP) quando da detecção de lesão coronariana proximal grave, a abordagem mais comum pelos centros de todos os grupos foi a realização de ICP antes da TAVI. Em casos considerados de risco para obstrução coronariana, os três grupos concordaram quanto à adoção de deixar um fio-guia de proteção na coronária durante a TAVI como estratégia mais comum (Tabela suplementar 4). Quanto à profilaxia antibiótica, mais de 90% dos centros administram antibióticos de rotina, sendo que metade deles administra 1 dose e a outra metade, pelo menos 2 doses.

### Manejo periprocedimento

**Figura 5:**
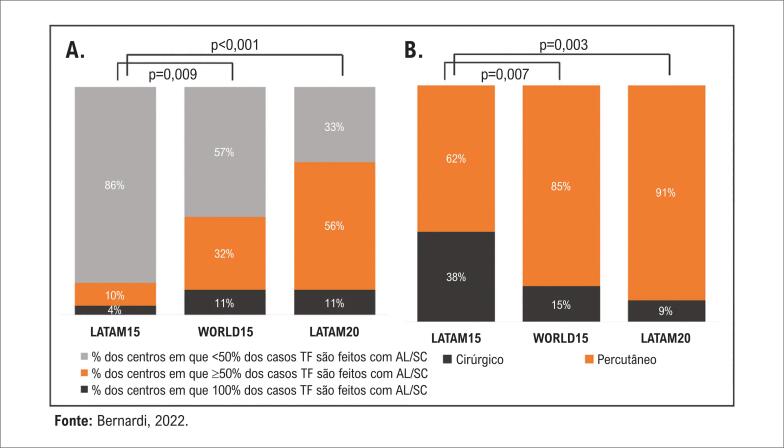
A) Porcentagens de procedimentos transfemorais realizados com sedação consciente / anestesia local (% de centros). TF: transfemoral; AL: anestesia local; SC: sedação consciente. B) Tipo de acesso vascular realizado rotineiramente para casos transfemorais (% de centros).

As comparações das respostas sobre manejo periprocedimento estão sumarizadas na Tabela 1. A abordagem transfemoral foi a mais empregada pelos centros dos três grupos, mas uma maior proporção de centros LATAM15 em relação a centros WORLD15 optou pelo acesso transfemoral em ≥ 90% dos seus casos (72% vs. 42%, respectivamente, p=0,003). Não houve alteração significativa após 5 anos (LATAM20 87%, p=0,14). Quase todos os centros reportaram a presença de um anestesiologista para auxiliar nos procedimentos transfemorais, mas os centros LATAM15 mais comumente realizaram esses procedimentos sob anestesia geral em comparação aos centros WORLD15 e LATAM20 (Figura 5). Além disso, 86% dos centros LATAM15 reportaram a presença de cirurgião cardíaco para auxiliar TAVI transfemoral vs. 61% dos centros WORLD15 (p=0,01) e 52% dos LATAM20 (p=0,005). Ainda, cardiologistas intervencionistas assistiram regularmente procedimentos transapicais/transaórticos na maioria dos centros LATAM15 (88%) e WORLD15 (88%), com uma significativa redução após 5 anos nos centros LATAM20 (56%, p=0,008). Com relação à utilização de ecocardiografia transesofágica para orientação do procedimento, 83% dos centros LATAM15 reportaram sempre utilizá-la em comparação a 41% dos centros WORLD15 e 15% dos LATAM20 (Tabela 1).

**Tabela 1:** Comparação das condutas técnicas periprocedimento entre os centros LATAM15, WORLD15 e LATAM20

	LATAM15 (N=29)	WORLD15 (N=221)	valor de p	LATAM20 (N=46)	valor de p#
**Locais onde a TAVI é realizada de rotina (% centros)**					
Sala cirúrgica	3%	9%	0,48	0	0,38
Laboratório de hemodinâmica	83%	63%	0,04	83%	1,0
Sala híbrida	24%	45%	0,04	19%	0,77
**ETE durante TAVI (% centros)**					
Sempre	83%	41%	<0,001	15%	<0,001
Apenas em certos pacientes	10%	42%		63%	
Nunca	7%	17%		22%	
**Tipo de dispositivo de oclusão usado de rotina no acesso percutâneo transfemoral (% centros)**					
1 Perclose	0	1%		9%	
2 ou mais Perclose	90%	59%	0,03	83%	0,17
Prostar	10%	40%		2%	
**Fio-guia de proteção da artéria contralateral nos casos percutâneos femorais (% centros)**					
Sempre	33%	35%	0,06	32%	
Nunca	4,8%	25,2%		4%	1,0
Apenas em acessos iliofemorais desafiadores	62%	40%		61%	
**Balão periférico durante fechamento do acesso nos casos percutâneos (% centros)**					
Rotineiramente	10%	12,9%	1,0	4%	0,6
Apenas em caso de complicação	90%	87,1%		96%	
**Se perfuração femoral nos casos percutâneos (% centros)**					
Em geral, implante de *stent* revestido autoexpansível ou balão-expansível	70%	78%	0,99	78%	0,54
Em geral, assistido por cirurgião vascular ou radiologista intervencionista	30%	22%		22%	
Dispositivo de proteção embólica de rotina (% centros)	0	16%	0,02	0	1,0

Observação:

# Valor de p dos centros LATAM20 em comparação aos resultados dos centros LATAM15.

TAVI: implante transcateter de válvula aórtica; ETE: ecocardiograma transesofágico; ETT: ecocardiograma transtorácico.

Nos casos transfemorais, TAVI com uma abordagem totalmente percutânea foi mais frequentemente realizada pelos centros WORLD15 e LATAM20 (Figura 5). Para esses, o dispositivo Perclose (Abbott Vascular, Abbott Park, IL) foi o mais utilizado em todos os grupos (Tabela 1). Quando perguntados sobre as estratégias de proteção no acesso percutâneo transfemoral, todos os grupos apontaram como estratégia mais frequente deixar um fio-guia de proteção contralateral apenas em casos de acesso iliofemoral mais desafiador e utilizar balão periférico durante o fechamento do acesso apenas na presença de complicação. No caso de perfuração femoral, a rotina mais comum consistiu no uso de *stent* revestido autoexpansível ou balão-expansível pelo próprio operador (Tabela 1).

A maioria dos centros dos três grupos relatou o uso regular do sistema Corevalve (Medtronic, Minneapolis, MN) e das válvulas Sapien (Edwards Lifesciences, Irvine, CA). Entretanto, em 2015 uma maior proporção de centros latino-americanos implantou uma válvula autoexpansível em > 50% dos seus pacientes em comparação aos centros do resto do mundo sem, sem uma alteração significativa deste cenário após 5 anos nos centros latino-americanos. Importante notar que, em 2015, apenas as válvulas cardíacas transcateter Corevalve e Sapien XT estavam comercialmente disponíveis na América Latina para essas famílias de próteses. Em contraste, na pesquisa LATAM20, a maioria dos centros usou os sistemas Evolut R e Sapien 3. Os centros WORLD15 utilizaram mais rotineiramente a valvoplastia com pré-dilatação do que os centros LATAM15 e LATAM20 (Tabela 2). Nem os centros LATAM15, nem os LATAM20 relataram o uso de dispositivos de proteção embólica como rotina em comparação a 16% dos centros WORLD15 (Tabela 1).

**Tabela 2:** Comparação entre os grupos do tipo de válvula implantada na TAVI

	LATAM15 (N=29)	WORLD15 (N=221)	valor de p	LATAM20 (N=46)	valor de p#
**Tipo de THV implantada de rotina (% centros)**					
Válvula Corevalve	86%	79%		91%	
Válvula Sapien	72%	84%		93%	
Válvula Acurate	10%	4%		41%	
Válvula Lotus	3%	26%		11%	
Válvula Portico	0	1%		0	
Centros onde >50% dos casos recebem THV autoexpansível (% centros)	52%	33%	0,06	46%	0,64
**Valvoplastia com pré-dilatação por balão de rotina (% centros)**					
Para válvulas auto-expansíveis	44%	50%	0,68	47%	0,81
Para válvulas balão-expansíveis	52%	68%	0,13	37%	0,23
Nenhum caso	30%	14%	0,04	44%	0,32

Observação:

# P-valor dos centros LATAM20 em comparação aos resultados dos centros LATAM15.

THV: válvula cardíaca transcateter.

### Manejo pós-procedimento e seguimento

Os principais achados do manejo pós-procedimento são apresentados na Tabela 3. A manutenção de telemetria pós-TAVI variou muito entre as instituições, sem diferença entre os centros LATAM15 e WORLD15 (72% vs. 59% durante 48 horas), embora tenha havido significativa redução nos centros LATAM20 (72% dos centros mantiveram telemetria por apenas 24 horas). Quando uma válvula autoexpansível foi implantada, os centros LATAM15 tenderam a remover o marca-passo temporário mais tarde do que os centros WORLD15 e LATAM20, não tendo sido observada diferença com as válvulas balão-expansíveis. A conduta inicial preferida no bloqueio atrioventricular transitório por todos os grupos foi manter o marca-passo temporário e observar, independentemente do tipo da válvula. Os centros também concordaram quanto à conduta em um novo bloqueio de ramo esquerdo, a maioria optando por manter a telemetria ou o marca-passo temporário por período mais longo enquanto se espera uma outra indicação de implante de marca-passo permanente (Tabela suplementar 5).

**Tabela 3:** Comparação das respostas relacionadas ao manejo pós-procedimento entre os centros LATAM15, WORLD15 e LATAM20

	LATAM15 (N=29)	WORLD15 (N=221)	valor de p	LATAM20 (N=46)	valor de p#
**Manutenção de telemetria após TAVI (% centro)**					
24h	36%	20%	0,13	72%	0,002
48h	36%	39%		24%	
>48h	28%	41%		4%	
**Manutenção de MPT após THV autoexpansível (na ausência de bloqueio AV ou de novo distúrbio de condução)**					
Remover sempre no final do procedimento	0	11%	0,004	24%	<0,001
Pelo menos 12-24h	30%	40%		59%	
Pelo menos 48h	59%	27%		4%	
Sem protocolo padrão	11%	22%		13%	
**Manutenção de MPT após THV balão-expansível (na ausência de bloqueio AV ou de novo distúrbio de condução)**					
Remover sempre no final do procedimento	71%	46%	0,08	70%	0,17
Pelo menos 12-24h	10%	24%		15%	
Pelo menos 48h	10%	6%		0	
Sem protocolo padrão	10%	24%		15%	
**Conduta para bloqueio AV transitório em THV autoexpansível (% centros)**					
Implantação direta de marca-passo permanente	4%	13%	0,31	7%	0,26
MPT e observação	81%	66%		63%	
A depender da existência de distúrbios de condução prévios	11%	14%		28%	
Outra	4%	6%		2%	
**Conduta para bloqueio AV transitório em THV balão-expansível (% centros)**					
Implantação direta de marca-passo permanente	4,5%	7%	0,06	4%	0,04
MPT e observação	87%	66%		63%	
A depender da existência de distúrbios de condução prévios	0	17%		26%	
Outra	9%	10%		2%	

Observação:

# Valor de p dos centros LATAM20 em comparação aos resultados dos centros LATAM15.

TAVI: implante transcateter de válvula aórtica; THV: válvula cardíaca transcateter; bloqueio AV: bloqueio atrioventricular; MPT: marca-passo temporário.

**Figura 6:**
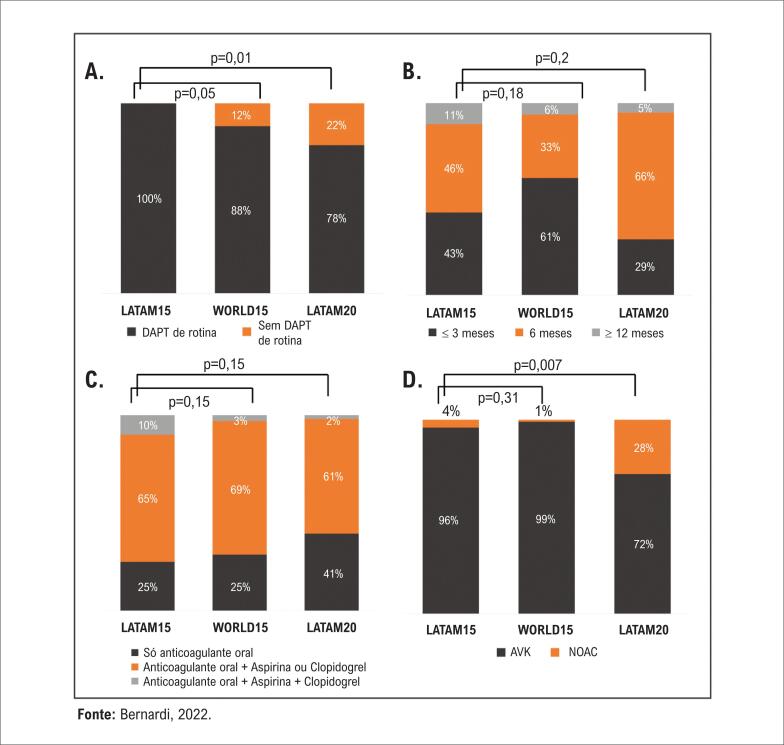
Terapia antitrombótica após TAVI. A) DAPT de rotina após TAVI na ausência de outra indicação para anticoagulação (% de centros). DAPT: terapia dupla antiplaquetária; B) Duração rotineira da DAPT (% de centros); C) Terapia antitrombótica rotineira em casos com indicação de anticoagulação (% de centros); D. Tipo de anticoagulante oral utilizado quando há indicação de anticoagulação (% de centros). AVK: antagonista da vitamina K; NOAC: novos anticoagulantes orais.

Quanto à terapia antitrombótica à alta hospitalar, na ausência de qualquer indicação de anticoagulação, DAPT com aspirina e clopidogrel foi a estratégia de escolha da maioria das instituições. Entretanto, nos últimos 5 anos, mais centros latino-americanos deram alta a seus pacientes em uso de apenas um agente antiagregante plaquetário (Figura 6). A duração da DAPT foi heterogênea, mas ~90% dos centros suspenderam um dos agentes em 6 meses. Nos pacientes com indicação de anticoagulantes, a terapia antitrombótica variou consideravelmente, sendo a associação de um anticoagulante oral com apenas um antiagregante plaquetário a opção preferida pela maioria dos centros em todos os grupos. Nesses casos, a utilização dos novos anticoagulantes orais (NOAC) aumentou significativamente de 4% para 28% nos centros latino-americanos no período de 5 anos (Figura 6).

## Discussão

No presente estudo, as práticas contemporâneas de TAVI nos centros latino-americanos e suas mudanças entre 2015 e 2020 foram avaliadas, tendo para comparação o *status* das práticas nos centros de países desenvolvidos em 2015. Os principais achados foram os seguintes: 1) no geral, os centros latino-americanos apresentaram experiência cumulativa e volume anual de procedimentos muito menores do que os centros do resto do mundo; 2) houve aumento na proporção de pacientes de risco cirúrgico baixo e intermediário sendo tratados com TAVI na América Latina; 3) a adoção de abordagens minimalistas de TAVI aumentou nos centros latino-americanos de 2015 a 2020, uma tendência já observada nos centros de países desenvolvidos em 2015; 4) o manejo pós-procedimento variou consideravelmente entre as instituições, mas algumas alterações significativas na prática foram observadas nos centros latino-americanos durante o período estudado, como redução no tempo de telemetria e da manutenção do marca-passo temporário após o procedimento, menor frequência de administração de DAPT na alta hospitalar e maior utilização de NOAC nos casos com recomendação clínica de anticoagulação.

### Volume por centro

Estudos recentes salientaram a importância do volume e da experiência do centro como indicadores na TAVI, relacionando-os a melhores desfechos clínicos e a mudanças nas técnicas/práticas de TAVI.^8–11^ No presente estudo, observamos que o volume de procedimentos nos centros latino-americanos é ainda inferior ao dos países desenvolvidos. Mesmo em 2020, a mediana do número de procedimentos realizados nas instituições latino-americanas correspondeu a um terço daquela nos centros do resto do mundo 5 anos antes. Nossos dados corroboram uma estimativa de 2017 sobre a dispersão geográfica da TAVI pelo mundo, mostrando que os países latino-americanos implantam, por 1 milhão de habitantes, menos de 10 válvulas, enquanto países como os Estados Unidos, França e Alemanha implantam mais de 100.^12^ Ao se considerar a proporção de números de centros por habitantes idosos, essa discrepância é ainda mais evidente. Atualmente, estima-se que existam 200 centros ativos de TAVI na América Latina para uma população idosa de ~56 milhões (3,6 centros/milhão) vs. 698 centros nos Estados Unidos para ~52 milhões de idosos (13,4 centros/milhão), de acordo com o Registro Nacional de Dados Cardiovasculares.^13,14^ Os fatores econômicos estão entre os motivos mais prováveis para explicar essa disparidade.

Nas últimas décadas, a despeito do crescimento econômico e da melhoria nos indicadores sociais, a desigualdade de riqueza persiste sendo um importante problema na América Latina, impactando de forma direta o bem-estar da população e os sistemas de saúde.^15^ Países em desenvolvimento em geral ficam atrás dos mais ricos quanto à implementação de procedimentos médicos de alta tecnologia e custo em seus sistemas de saúde, o que é o caso da TAVI e da cirurgia cardiovascular em geral.^16^ Com as alterações demográficas na América Latina tendendo ao envelhecimento populacional, espera-se que a demanda por TAVI aumente. Para que os sistemas de saúde deem conta dessa demanda, os governos e líderes locais precisam encontrar maneiras de melhorar a custo-efetividade da TAVI no continente. A implementação de políticas para a redução dos custos dos procedimentos é fundamental, primeiramente ao reduzir os preços dos dispositivos que hoje representam, em média, ~70% do custo total do procedimento. Isso poderia ser obtido com o subsídio ou redução das taxas de importação, estimulando a vinda de mais indústrias médicas para a América Latina e criando incentivos para a fabricação local de próteses de alto custo, o que vem acontecendo no Brasil recentemente. Quanto à efetividade, o presente estudo sinaliza para a redução nas disparidades quanto às práticas atuais de TAVI entre países latino-americanos e do restante do mundo. Além disso, dados do registro brasileiro de TAVI de 2016 mostram desfechos clínicos em concordância com a literatura.^17^ Essas boas práticas se devem principalmente a um importante apoio das sociedades e indústrias médicas locais, promovendo sessões científicas, treinamentos práticos e programas intensivos de *proctoring* na América Latina nos últimos anos.

### Manejo periprocedimento

Além da relação volume-desfecho, existe uma relação volume-prática, pois os centros com maior volume de TAVI alteram suas práticas rotineiras ao longo do tempo. Uma análise recente do registro norte-americano de terapia valvar transcateter (TVT) sobre a curva de aprendizado de TAVI demonstra que, à medida que a experiência cumulativa da instituição progride, aumenta a probabilidade de os procedimentos de TAVI serem realizados com sedação consciente, anestesia local e acesso vascular totalmente percutâneo, a chamada abordagem minimalista.^8,11^ A despeito da falta de dados definitivos na literatura mostrando que essas técnicas menos invasivas estão diretamente associadas com melhora nos desfechos clínicos duros,^18–21^ com certeza elas representam um avanço na *expertise* desses ‘*heart teams*’.

O presente estudo capturou este fenômeno. Em 2015, uma maior proporção de centros ao redor do mundo já havia adotado o uso rotineiro da TAVI minimalista em comparação aos centros latino-americanos. Interessante notar que, após 5 anos, embora os centros latino-americanos continuem com baixos volumes de procedimentos em geral, com mediana de apenas 16 casos por ano, essas técnicas mais atuais vêm sendo consistentemente incorporadas. A proporção de centros que realizaram mais de metade dos casos com anestesia local e sedação consciente aumentou cerca de seis vezes. Tendência similar foi observada no registro TVT nos últimos anos, com relato de aumento consistente dos procedimentos com sedação consciente, atualmente correspondendo a 64% dos casos da América do Norte.^22^ Da mesma forma, abordagem totalmente percutânea como prática rotineira aumentou de 62% para 91% nos centros latino-americanos, mostrando que a prática de TAVI está evoluindo no continente a despeito das limitações para aumentar o volume de procedimentos.

### Manejo pós-procedimento e seguimento

O manejo pós-procedimento adequado é outro fator fundamental, ainda que por vezes negligenciado, num programa de TAVI. Importante salientar que, a maioria dos ensaios clínicos até o momento visou avaliar aspectos do procedimento de TAVI. Consequentemente, há escassez de dados definitivos sobre a melhor conduta em relação aos pacientes após o procedimento. Não é de surpreender que o presente estudo mostre heterogeneidade na prática entre os centros nesse aspecto. No entanto, algumas mudanças significativas na prática foram observadas nos centros latino-americanos nos últimos 5 anos. Houve menos prescrição rotineira de DAPT na alta hospitalar e utilização mais frequente de NOAC para pacientes com indicação de anticoagulação. Tais mudanças na prática podem ser atribuídas a dados publicados entre as duas pesquisas mostrando o potencial benefício da terapia antiplaquetária oral única na redução de complicações de sangramento^23^ e ao uso mais disseminado de NOAC na cardiologia em geral, devido ao melhor perfil de segurança nos pacientes idosos. No entanto, o regime antitrombótico ótimo e a utilização de NOAC após TAVI permanecem em debate, particularmente após os resultados desanimadores de um grande ensaio randomizado recente testando a rivaroxabana no pós-TAVI.^24^ Portanto, dados de ensaios clínicos randomizados futuros são necessários para definir o melhor manejo pós-procedimento.

Por fim, a progressão das práticas de TAVI na América Latina revela que mesmo os centros dos países em desenvolvimento e com menores volumes podem acompanhar a rápida evolução nessa área. Isso tem sido catalisado graças ao grande engajamento das sociedades médicas na disseminação do conhecimento na América Latina. No Brasil, por exemplo, adotou-se uma certificação formal de TAVI desde 2017. Através de programas educacionais multifacetados e em vários níveis, o país já treinou mais de 700 cardiologistas. Da mesma forma, iniciativas similares foram adotadas em outros países, como Argentina, Chile, Colômbia e México. Todos esses esforços contribuíram para o aumento do número de novos centros que realizam TAVI na América Latina e desempenharam papel significativo no desenvolvimento das técnicas mais modernas e na adesão às mesmas. Entretanto, muito há que se fazer para que se continue a diminuir a lacuna em relação aos países desenvolvidos. À medida que aumenta o número de centros de TAVI, será necessário expandir a supervisão e fornecer programas de educação médica continuada. Na era pós-COVID-19, inovações, como telemonitoramento, podem ter valor inestimável. A criação de programas de simulação virtual para reduzir a curva de aprendizado dos centros/operadores de baixo volume parece outra opção atraente.^25^ Por fim, melhorar a publicação de conteúdo científico pelos centros latino-americanos se faz necessário, além da criação de bancos de dados de abrangência nacional nos países latino-americanos para determinar os reais desfechos clínicos e definir as potenciais oportunidades de melhoria.

### Limitações

Embora este estudo tenha sido uma oportunidade única para capturar variações na prática entre centros e regiões do mundo, assim como as mudanças nos centros latino-americanos nos últimos 5 anos, algumas limitações devem ser mencionadas. Primeiramente, este foi um estudo baseado numa pesquisa voluntária autorrelatada, estando propenso a viés. Seus resultados podem sub- ou superestimar a verdadeira realidade dos centros participantes. Relatos sobre as diferenças nas características de base dos pacientes tratados em cada centro, que poderiam influenciar a adoção de práticas diferentes, não estavam disponíveis. Além disso, o estudo não incluiu informação sobre desfechos clínicos. Portanto, não se pode concluir se as diferenças na prática impactaram os desfechos dos pacientes. Ademais, há grande heterogeneidade entre os países, as regiões e as instituições latino-americanas. É difícil supor que uma pesquisa possa representar com precisão a realidade de todo o continente, a despeito da estimativa de que ~15% do total de centros latino americanos de TAVI participaram da última pesquisa. De toda forma, os resultados do presente manuscrito indicam a direção em que seguimos e as lacunas que ainda precisam ser preenchidas, além de servirem como guia para os centros de menor experiência na definição de seus protocolos institucionais. Por fim, como a pesquisa WRITTEN não foi reconduzida no resto do mundo no período 2019-2020, não foi possível fazer uma comparação direta da prática de TAVI atual na América Latina com a de outros centros.

## Conclusão

Em conclusão, diferenças referente às práticas de TAVI existem entre os países latino americanos e países mais desenvolvidos, havendo um atraso de pelo menos 5 anos na adoção disseminada de algumas técnicas mais modernas na América Latina. Algumas dessas diferenças na prática de TAVI parecem ligadas ao menor volume de procedimentos nos centros latino-americanos, enquanto outras podem estar meramente associadas à falta de consenso global e variabilidade regional. Entretanto, a lacuna parece estar diminuindo, pois a relação volume de procedimentos/desenvolvimento das práticas de TAVI tem se transformado nos últimos anos através da adoção de técnicas mais refinadas mesmo pelos centros latino-americanos de menor volume. De toda maneira, estudos futuros no continente serão necessários para avaliar o impacto de tais mudanças na prática sobre os desfechos clínicos dos pacientes.

## *Material suplementar

Para informação adicional, por favor, clique aqui.
