# Supplementary material for: Evolução e Estado Atual das Práticas de Implante Transcateter de Válvula Aórtica na América Latina – Estudo WRITTEN LATAM
Source: Arq Bras Cardiol. 2022 Jun 6;118(6):1085–96. [Article in Portuguese] doi: 10.36660/abc.20210327 (PMC9345155; doi:10.36660/abc.20210327)
Supplement: Translation

# Translation: Recent Developments and Current Status of Transcatheter Aortic Valve Replacement Practice in Latin America – the WRITTEN LATAM Study

**Authors:** Fernando Luiz de Melo Bernardi, Henrique Barbosa Ribeiro, Luis Nombela-Franco, Enrico Cerrato, Gabriel Maluenda, Tamim Nazif, Pedro Alves Lemos, Matias Sztejfman, Pablo Lamelas, Dario Echeverri, Marcelo Antonio Cartaxo Queiroga Lopes, Fábio Sândoli de Brito, Alexandre A. Abizaid, José A. Mangione, Helene Eltchaninoff, Lars Søndergaard, Josep Rodes-Cabau

**Affiliations:** 1 São Paulo SP Brazil Universidade de São Paulo – Faculdade de Medicina Hospital das Clínicas – Instituto do Coração, São Paulo, SP – Brazil; 2 São Paulo SP Brazil Hospital Samaritano Paulista, São Paulo, SP – Brazil; 3 São Paulo SP Brazil Hospital Sírio-Libanês – Hemodinâmica e Cardiologia Intervencionista, São Paulo, SP – Brazil; 4 Madrid Spain Hospital Clínico San Carlos, Madrid – Spain; 5 Orbassano Italy San Luigi Gonzaga University Hospital, Orbassano – Italy; 6 Santiago Chile Hospital Clínico San Borja Arriarán, Santiago – Chile; 7 New York USA Columbia University Medical Center, New York – USA; 8 São Paulo SP Brazil Hospital Israelita Albert Einstein, São Paulo, SP – Brazil; 9 Buenos Aires Argentina Sanatorio Finochietto, Buenos Aires – Argentina; 10 Buenos Aires Argentina Instituto Cardiovascular de Buenos Aires, Buenos Aires – Argentina; 11 Bogota Colombia Fundacion Cardioinfantil Instituto de Cardiologia, Bogota, Cundinamarca – Colombia; 12 João Pessoa PB Brazil Hospital Alberto Urquiza Wanderley, Cabedelo, João Pessoa, PB – Brazil; 13 São Paulo SP Brazil Beneficência Portuguesa de São Paulo, São Paulo, SP – Brazil; 14 Rouen Normandy France Hôpital Charles Nicolle, Rouen, Normandy – France; 15 Kobenhavn Denmark Copenhagen University Hospital, Kobenhavn – Denmark; 16 Quebec Canada Institut Universitaire de Cardiologie Et de Pneumologie de Québec, Quebec – Canada

**Keywords:** Transcatheter Aortic Valve Replacement, Aortic Valve Stenosis, Latin America

## Abstract

**Background:**

Transcatheter aortic valve replacement (TAVR) is a worldwide adopted procedure with rapidly evolving practices. Regional and temporal variations are expected to be found.

**Objective:**

To compare TAVR practice in Latin America with that around the world and to assess its changes in Latin America from 2015 to 2020.

**Methods:**

A survey was applied to global TAVR centers between March and September 2015, and again to Latin-American centers between July 2019 and January 2020. The survey consisted of questions addressing: i) center’s general information; ii) pre-TAVR evaluation; iii) procedural techniques; iv) post-TAVR management; v) follow-up. Answers from the 2015 survey of Latin-American centers (LATAM15) were compared with those of other centers around the world (WORLD15) and with the 2020 updated Latin-American survey (LATAM20). A 5% level of significance was adopted for statistical analysis.

**Results:**

250 centers participated in the 2015 survey (LATAM15=29; WORLD15=221) and 46 in the LATAM20. Combined centers experience accounted for 73 707 procedures, with WORLD15 centers performing, on average, 6- and 3-times more procedures than LATAM15 and LATAM20 centers, respectively. LATAM centers performed less minimalistic TAVR than WORLD15 centers, but there was a significant increase in less invasive procedures after 5 years in Latin-American centers. For postprocedural care, a lower period of telemetry and maintenance of temporary pacing wire, along with less utilization of dual antiplatelet therapy was observed in LATAM20 centers.

**Conclusion:**

Despite still having a much lower number of procedures, many aspects of TAVR practice in Latin-American centers have evolved in recent years, followingthe trend observed in developed country centers.

## Introduction

Transcatheter aortic valve replacement (TAVR) has been adopted worldwide for severe symptomatic aortic stenosis with various risk profiles. This achievement has been built on more than a decade of advancements in technology and patient care. As a consequence, TAVR practices have been evolving rapidly, resulting in a significant improvement in clinical outcomes.^1–4^

In Latin America, the first TAVR procedures were performed in 2008 in Brazil and Colombia.^5,6^ Although a steady growth of cases has been observed since then, there have been concerns in the adoption of the most up-to-date practices in Latin America.^8–10^ In developing countries, disparities in practice of a high-cost medical procedure can be exacerbated due to several factors, such as lower-income health systems, lower center volumes, less experienced operators, unavailability of certain devices, among others. Understanding such differences is crucial to better comprehend the contemporary practices and seek for further standardization. Moreover, it could aid in developing policies by the local regulators to achieve more widespread adoption of TAVR in such underserved populations, since published data in Latin America are limited.

Therefore, the general and secondary objectives of the study were: i) to compare TAVR practice between Latin-American centers and centers from the rest of the world based on data obtained from the 2015 WRITTEN survey; ii) to assess the changes in TAVR practice in Latin America after 5 years through reapplication of the survey in the continent.

## Methods

The WRITTEN survey was an internet-based questionnaire designed to investigate the practices in TAVR centers around the world. The survey design has been described previously.^7^ In summary, at least one regional TAVR expert from each country or region was contacted and invited to distribute the survey locally. The survey was promoted through general interventional cardiology mailing lists, announcements by official societies of interventional cardiology, website advertisements, and personalized emails to TAVR operators. Invitations were distributed in different geographic areas simultaneously over 6 months (March 2015 to September 2015). A second enquiry was performed from July 2019 to January 2020, with similar methods, involving only Latin-American centers without a specific cutoff on the number of procedures performed by the center (Figure 1). The survey consisted of an online platform hosted on the collaborative research website (www.cardiogroup.org/TAVI/) with 59 questions addressing five domains of TAVR (Supplemental Table 1): (i) general information about the program at each institution, (ii) patient selection, (iii) procedural techniques and imaging, (iv) postprocedural management, and (v) follow-up. It was requested that only one individual from each TAVR center completed the survey, and only one questionnaire per center was accepted.

**Figure 1:** Geographical distribution of the participating centers in the 2015 and 2020 surveys.

### Statistical analysis

For the study analysis, the answers corresponding to the TAVR practices of the Latin-American centers in 2015 (LATAM15 centers) were used as reference. Categorical variables were expressed as absolute frequencies and percentages, and continuous variables as median and interquartile range (IQR). For comparison of categorical variables, Fisher’s exact test was used to assess the association between dependent (centers group) and independent variables (results from the questionnaire) for dichotomous answers with a two-tailed P value. For questions with more than two possible answers, the association between independent and dependent variables was tested with the chi-square test. Continuous variables were compared with the Mann-Whitney test due to the non-normal distribution of the variables, confirmed by the Shapiro-Wilk test, also with a two-tailed P value. A 5% level of significance was adopted for all statistical analyses. All analyses were performed with the software GraphPad Prism version 7.0 (GraphPad Software, USA).

## Results

As previously published, 250 centers completed the questionnaire properly and were included in the 2015 survey.^7^ Of these, 29 (11.6%) were from LATAM15 centers. Figure 1 illustrates the global distribution of the centers. Figure 2 summarizes the enrollment of the 46 centers participating in the Latin-American survey in 2020 (LATAM20). Out of the 296 questionnaires, 263 (88.8%) were fully answered, while the remaining had more than 80% of their questions responded. The very few missing data were considered as completely at random, and no special treatment was made. The names of the cities and countries of all centers are listed in the Supplemental Tables 2 and 3.

**Figure 2:** Enrollment flowchart of the 2020 WRITTEN LATAM survey.

By the time of the surveys’ completion, the sum of all TAVR performed by the participating centers in Latin America in 2015 and 2020 (LATAM15 and LATAM20) and worldwide (WORLD) accounted for 73 707 procedures combined. In comparison to LATAM15, WORD15 centers had performed a much higher number of procedures in their whole experience (median of 34, IQR: 12 to 101 vs. 200, IQR: 84 to 453, p<0.001), as well as in the year before survey completion (median of 12, IQR: 5 to 23 vs. 60, IQR: 27 to 110, p<0.001). Compared to LATAM15, the LATAM20 total experience was ~2-fold larger (median of 62, IQR: 22 to 138, p=0.08), but only slightly higher in the year before the survey (median of 16, IQR: 6 to 30, p=0.29). The complete survey results are found in Supplemental Tables 4-7.

### Pre-procedural evaluation

In all three groups, the majority of TAVR patients treated in their current practice were at high or prohibited surgical risk. Nonetheless, when comparing LATAM15 to LATAM20, an increase over time was observed in the proportion of intermediate and low surgical risk patients (Figure 3). WORLD15 centers had a higher median number of heart-team meetings monthly than LATAM15 centers (4, IQR: 2 to 4 vs. 1, IQR: 1 to 2, p=0.001), with a slight increase in LATAM20 centers (1.5, IQR: 1 to 4, p=0.27). The Society of Thoracic Surgeons (STS) score was the most common risk-stratification tool, used routinely by 90%, 69%, and 98% of the LATAM15, WORDL15, and LATAM20 centers, respectively. Meanwhile, only 28%, 47%, and 39% of the centers, respectively, applied frailty tests routinely. Regarding pre-TAVR imaging (Figure 4), almost all centers performed cardiac computed tomography in their practice. Transesophageal echocardiography as a routine before the procedure was performed more often by LATAM15 centers.

**Figure 3:** Mean proportions of treated patients according to the risk profile.

**Figure 4:** Routinely performed preprocedural imaging studies (% of centers). TEE: transesophageal echocardiogram; CT: computed tomography

A lower proportion of WORLD15 and LATAM20 centers regularly administered dual-antiplatelet therapy (DAPT) before transfemoral procedures in comparison to LATAM15 centers (45% and 56% vs. 83%, p<0.001 and p=0.02, respectively). Regarding the time of percutaneous coronary intervention (PCI) when a severe proximal coronary lesion was detected, the most common approach by the centers from all groups was to perform PCI before TAVR. In cases deemed risky for coronary obstruction, the three groups agreed the most frequent strategy was to have a PCI protection wire during TAVR (Supplemental Table 4). Regarding antibiotic prophylaxis, more than 90% of the centers administer antibiotics as a routine, with half of them administering 1 dose and the other half ≥ 2 doses.

### Procedural management

The comparison of answers to procedural management questions is summarized in Table 1. Transfemoral TAVR was the preferred approach by all centers, but a higher proportion of LATAM15 over WORLD15 centers performed ≥ 90% of their cases via the transfemoral route (72% vs. 42%, respectively, p=0.003). No significant change was noted after 5 years (LATAM20 87%, p=0.14). Almost all centers reported having an anesthesiologist to assist in transfemoral procedures, but LATAM15 centers more commonly performed these procedures under general anesthesia compared to WORLD15 and LATAM20 centers (Figure 5). Additionally, 86% of LATAM15 centers reported having a cardiac surgeon assisting transfemoral TAVR vs. 61% for WORLD15 (p=0.01) and 52% for LATAM20 (p=0.005). Meanwhile, interventional cardiologists regularly assisted transapical/transaortic procedures in most LATAM15 (88%) and WORLD15 (88%) centers, with a significant reduction after 5 years in LATAM20 centers (56%, p=0.008). Regarding procedural transesophageal echocardiography guidance, 83% of LATAM15 centers reported always relying on it, compared to 41% for WORLD15 and 15% for LATAM20 centers (Table 1).

**Table 1:** Comparison of technical procedural management between the LATAM15, WORLD15, and LATAM20 centers

	LATAM15 (N=29)	WORLD15 (N=221)	p value	LATAM20 (N=46)	p value#
**Site where TAVR is routinely performed (% centers)**					
Operating room	3%	9%	0.48	0	0.38
Cath lab	83%	63%	0.04	83%	1.0
Hybrid room	24%	45%	0.04	19%	0.77
**TEE during TAVR (% of centers)**					
Always	83%	41%	<0.001	15%	<0.001
Only in certain patients	10%	42%		63%	
Never	7%	17%		22%	
**Type of closure device routinely used in transfemoral percutaneous access (% centers)**					
1 Perclose	0	1%		9%	
2 or more Perclose	90%	59%	0.03	83%	0.17
Prostar	10%	40%		2%	
**Protection guidewire from contralateral artery in femoral percutaneous cases (% of centers)**					
Always	33%	35%	0.06	32%	
Never	4.8%	25.2%		4%	1.0
Only in challenging iliofemoral access	62%	40%		61%	
**Peripheral balloon during access closure in percutaneous cases (% centers)**					
Routinely	10%	12.9%	1.0	4%	0.6
Just in case of complication	90%	87.1%		96%	
**In case of femoral perforation in percutaneous cases (% centers)**					
Usually implant self-expandable or balloon-expandable covered stent	70%	78%	0.99	78%	0.54
Usually assisted by vascular surgeons or an interventional radiologist	30%	22%		22%	
Embolic protection device as a routine (% centers)	0	16%	0.02	0	1.0

Notes:

# P-values for the LATAM20 in comparison to the LATAM15 results.

TAVR: transcatheter aortic valve replacement; TEE: transesophageal echocardiography; TTE: transthoracic echocardiography.

**Figure 5:** A) Percentages of transfemoral procedures performed with conscious sedation/local anesthesia (% of centers). TF: transfemoral; LA: local anesthesia; CS: conscious sedation. B) Type of vascular access routinely performed for transfemoral cases (% of centers).

In transfemoral cases, TAVR with a fully percutaneous approach was more frequently performed by the WORLD15 and LATAM20 centers (Figure 5). For these, the Perclose (Abbott Vascular, Abbott Park, IL) was the most utilized device in all groups (Table 1). When asked about protective strategies in percutaneous transfemoral access, the most common approach by all groups was to leave a protection guidewire from the collateral artery only in challenging iliofemoral access and use of a peripheral balloon during access closure only when a complication ensues. In the case of femoral perforation, the most common approach consisted of using self- or balloon-expandable covered stent by the operator himself (Table 1).

The Corevalve system (Medtronic, Minneapolis, MN) and Edwards valves (Edwards Lifesciences, Irvine, CA) were reported as being regularly used by most centers from all three groups. Nonetheless, in 2015 a higher proportion of Latin-American centers implanted a self-expanding valve in > 50% of their patients compared to the other centers in the world without a significant change after 5 years in Latin-American centers. Of note, in 2015, only the Corevalve and Sapien XT transcatheter heart valves were commercially available in Latin America for these families of valves. In contrast, for LATAM20, most centers used the Evolut R and the Sapien 3 systems. The WORLD15 centers more routinely employed predilatation valvuloplasty than LATAM15 and LATAM20 centers (Table 2). Neither LATAM15 nor LATAM20 centers reported using embolic protection devices as a routine as compared to 16% of the WORLD15 centers (Table 1).

**Table 2:** Comparison of the type of transcatheter heart valve implanted between groups

	LATAM15 (N=29)	WORLD15 (N=221)	p value	LATAM20 (N=46)	p value#
**Type of THV routinely implanted (% centers)**					
Corevalve system	86%	79%		91%	
Edwards valve	72%	84%		93%	
Acurate valve	10%	4%		41%	
Lotus valve	3%	26%		11%	
Portico valve	0	1%		0	
Centers where >50% of cases are done with self-expanding THV (% centers)	52%	33%	0.06	46%	0.64
**Routine balloon predilatation valvuloplasty (% centers)**					
For self-expanding valves	44%	50%	0.68	47%	0.81
For balloon-expandable valves	52%	68%	0.13	37%	0.23
In no case	30%	14%	0.04	44%	0.32

Notes:

# P-values for the LATAM20 in comparison to the LATAM15 results.

THV: transcatheter heart valve.

### Postprocedural management and follow-up

The main findings on postprocedural care are shown in Table 3. Maintenance of telemetry after TAVR varied widely among institutions, with no difference between LATAM15 and WORLD15 centers (72% vs. 59%, during 48 hours), although a significant reduction in the period of surveillance was observed in LATAM20 centers (72% of centers maintained telemetry for just 24 hours). When a self-expandable valve was implanted, LATAM15 centers tended to remove the temporary pacemaker wire (TPW) later than WORLD15 and LATAM20 centers, whereas no difference was seen with balloon-expandable valves. The preferred initial management of transient atrioventricular block by all groups was to keep the TPW and watch, regardless of the type of valve. Centers also agreed on the management of a new left bundle branch block, most opting to keep telemetry or TPW for a longer period while waiting for any other indication of permanent pacemaker implantation (Supplemental Table 5).

**Table 3:** Comparison of answers regarding postprocedural care between LATAM15, WORLD15, and LATAM20 centers

	LATAM15 (N=29)	WORLD15 (N=221)	p value	LATAM20 (N=46)	p value#
**Maintenance of telemetry after TAVR (% center)**					
24h	36%	20%	0.13	72%	0.002
48h	36%	39%		24%	
>48h	28%	41%		4%	
**Maintenance of TPW after self-expanding THV (if no AV block or new conduction disorder)**					
Always remove at the end of procedure	0	11%	0.004	24%	<0.001
At least 12-24h	30%	40%		59%	
At least 48h	59%	27%		4%	
No standardized protocol	11%	22%		13%	
**Maintenance of TPW after balloon-expandable THV (if no AV block or new conduction disorder)**					
Always remove at the end of procedure	71%	46%	0.08	70%	0.17
At least 12-24h	10%	24%		15%	
At least 48h	10%	6%		0	
No standardized protocol	10%	24%		15%	
**Management of transient AV block in self-expanding THV (% centers)**					
Direct permanent pacemaker implantation	4%	13%	0.31	7%	0.26
TPW and watch	81%	66%		63%	
Depends on existence of prior conduction disorders	11%	14%		28%	
Other	4%	6%		2%	
**Management of transient AV-block in balloon-expandable THV (% centers)**					
Direct permanent pacemaker implantation	4.5%	7%	0.06	4%	0.04
TPW and watch	87%	66%		63%	
Depends on existence of prior conduction disorders	0	17%		26%	
Other	9%	10%		2%	

Notes:

# P-values for the LATAM20 in comparison to the LATAM15 results.

TAVR: transcatheter aortic valve replacement; THV: transcatheter heart valve; AV-block: atrioventricular block; TPW: temporary pacing wire.

Concerning the antithrombotic therapy at discharge, when no indication for anticoagulation existed, DAPT with aspirin and clopidogrel was the strategy of choice for most institutions. However, within the past 5 years, more Latin-American centers discharged their patients with a single antiplatelet agent (Figure 6). For the duration of DAPT, there was heterogeneity in practice, but ~90% of the centers suspended one of the agents within 6 months. In patients with an indication for anticoagulants, antithrombotic therapy varied considerably, being the association of an oral anticoagulant with only one antiplatelet agent the preferred choice by most centers from all groups. In these cases, the utilization of novel oral anticoagulants (NOACs) increased significantly from 4% to 28% in Latin-American centers during the 5-year period (Figure 6).

**Figure 6:** Antithrombotic therapy after TAVR. A) Routine DAPT after TAVR when no other indication for anticoagulation exists (% of centers). DAPT: dual-antiplatelet therapy; B) Routine duration of DAPT (% of centers); C) Routine antithrombotic therapy in cases where there is an indication for anticoagulation (% of centers); D. Type of oral anticoagulant utilized when an indication for anticoagulation exists (% of centers). VKA: vitamin K antagonist; NOAC: novel oral anticoagulant.

## Discussion

In the present study, the current TAVR practices in Latin-American centers and their changes between 2015 and 2020 were evaluated, having for comparison the practice status at centers from developed countries in 2015. The main findings can be summarized as: 1) overall, Latin-American centers had a much lower cumulative experience and annual volume in comparison to centers from the rest of the world; 2) there has been an increase in the proportion of low and intermediate surgical risk patients now being treated with TAVR in Latin America; 3) the adoption of minimalistic TAVR approaches has increased in Latin-American centers from 2015 to 2020, a trend already observed in centers around the world in 2015; 4) postprocedural care varied considerably among institutions, but some significant changes in the TAVR practice have been observed in Latin-American centers over the studied period, such as a reduction in the time of telemetry and TPW after the procedure, less frequent administration of DAPT, and more frequent use of NOACs when anticoagulation was clinically recommended.

### Center volume

Recent studies have highlighted the importance of center volume and experience as indicators in TAVR, linking them to improved outcomes and better practices.^8–11^ In the present study, we observed that the volume of procedures in Latin-American centers is still much lower than that in developed countries. Even in 2020, the median number of procedures performed in Latin-American institutions corresponded to a third of the volume performed in centers around the world 5 years earlier. Our data corroborate an estimate from 2017 on the geographical dispersion of TAVR across the world, showing that Latin-American countries implant less than 10 valves per 1 000 000 inhabitants, while the numbers for nations, such as the United States, France, and Germany, were above 100 implants per 1 000 000 people.^12^ When considering the proportion of centers per elderly inhabitants, this discrepancy is even more evident. Currently, Latin America has an estimate of 200 active TAVR centers for an elderly population of ~56 million (3.6 centers/million) vs. 698 centers in the United States (according to the National Cardiovascular Data Registry^13^) for ~52 million elderly (13.4 centers/million).^14^ Economic factors are most probably one of the most significant in explaining this disparity.

Over the past decades, despite economic growth and improvement in social indicators, wealth inequality is still a major issue in Latin America, directly impacting population well-being and health systems.^15^ Developing countries often lag behind wealthier nations in implementing high-cost technological medical procedures in their health systems, which is the case of TAVR and cardiovascular surgery in general.^16^ With demographic changes in Latin America towards population aging, the demand for TAVR is expected to rise accordingly. For the health systems to afford such demand, governments and local leaders will need to find ways to improve the cost-effectiveness of TAVR in the continent. Implementation of policies targeting a reduction in procedural costs will be key, primarily by lowering device prices that today represent on average ~70% of the procedure’s total cost. This could be achieved by subsidizing or reducing importation taxes, stimulating more medical industries to come to Latin America, and creating incentives for manufacturing the high-cost prosthesis locally, which has been the case of Brazil recently. On the effectiveness side, the present study signals to a reduction in the disparities between Latin-American countries and the current TAVR practices compared to the rest of the world. In addition, data from the Brazilian TAVR registry from 2016 showed similar clinical outcomes as compared with the literature, even though more contemporary data is lacking.^17^ This development in practice can be attributed mainly to a strong support of the local medical societies and industries, promoting scientific and hands-on training sessions, along with strong proctoring programs in Latin America over the recent years.

### Periprocedural management

In addition to a volume-outcomes relationship, a volume-practice relationship exists, as centers with a higher number of TAVR change their routine practice over time. Recent analysis from the North American Transcatheter Valve Therapy (TVT) Registry on the TAVR learning curve demonstrates that, as an institution’s cumulative experience progresses, TAVR procedures are more likely to be performed with conscious sedation, local anesthesia, and fully percutaneous vascular access. The so-called minimalistic approach.^8,11^ Although there is no definitive data in the literature showing that these less invasive techniques are directly associated with improvements in hard clinical outcomes,^18–21^ they surely represent incremental expertise of the heart teams.

The present study captured this phenomenon. In 2015, a higher proportion of centers around the world had already adopted the routine use of the minimalistic TAVR when compared to their Latin-American counterparts. But interestingly, after 5 years, even though Latin-American centers continue to have low volumes overall, with a median of only 16 cases yearly, there has been consistent incorporation of these more current techniques. The proportion of centers that performed more than half of cases with local anesthesia and conscious sedation increased ~6-fold. A similar trend has been observed in the TVT Registry during the latest years, where a steady increase in conscious sedation procedures has been reported, currently accounting for 64% of the North American cases.^22^ Similarly, a fully percutaneous approach as a routine practice increased from 62% to 91% of the Latin-American centers, showing that TAVR practices are evolving in the continent despite the struggle to improve procedural volume.

### Postprocedural management and follow-up

Proper postprocedural care is another fundamental, but sometimes overlooked, factor in a TAVR program. Of note, most clinical trials to date have aimed to assess intraprocedural aspects of TAVR. Consequently, there is a scarcity of definitive data on the best management of patients after the procedure. Not surprisingly, the present study showed heterogeneity in practice among centers in this domain. Yet, some significant changes in practice have been noted in Latin-American centers in the last 5 years. The routine prescription of DAPT on hospital discharge was less frequent and NOACs were more often used in patients with an indication for oral anticoagulation therapy. These changes in practice are probably attributed to data published between the two surveys showing a potential benefit of single oral antiplatelet therapy in reducing bleeding complications^23^ and to a more widespread use of NOACs in general cardiology due to safety profile in elderly patients. Still, the optimal antithrombotic regimen and the utilization of NOACs after TAVR remain open to debate, particularly after the dismal results from a recent large randomized trial with rivaroxaban.^24^ Hence, data from future randomized trials are warranted to define the optimal postprocedural care.

Finally, the progression of Latin-American practices reveals that even centers from developing and underserved countries can follow along with the rapid ongoing progressions in the field. This has been catalyzed thanks to a deep engagement of the medical societies in spreading the knowledge in Latin America. For instance, in Brazil, a formal TAVR certification has been adopted since 2017. Through multi-faceted and multilevel educational programs, the country has already trained more than 700 cardiologists. Likewise, similar initiatives in other countries, such as Argentina, Chile, Colombia, and Mexico, have also been adopted. All these efforts have contributed to a steady increase in new centers performing TAVR in Latin America and have played a significant role in the development of the most modern techniques and adherence to them. However, continous efforts should be implemented for diminishing the gap to developed nations. As the number of TAVR centers increases, expansion of proctoring and continuing medical education programs will be necessary. In the post-COVID-19 era, innovations, like teleproctoring, can be an invaluable asset. The creation of virtual simulation programs to soften the learning curve of lower volume centers/operators seems another attractive emerging option.^25^ Finally, improving publication of scientific content by Latin-American centers is urgently warranted, accompanied by the creation of nationwide databanks in all Latin-American countries to determine the actual clinical outcomes and further define the potential gaps for improvement.

### Limitations

Although this study was a unique opportunity to capture variations in practice among centers and regions of the world, as well as the changes in Latin-American centers over the past 5 years, some limitations must be mentioned. First, this was a self-reported voluntary survey, which, by its nature, makes it prone to biases. Results from such studies can under- or overestimate the actual reality of the participating centers. Reports on the differences in the baseline characteristics of the patients treated by each center, which could influence the adoption of different practices, were not available. Moreover, the study did not include information on clinical outcomes. Thus, it is impossible to draw conclusions on whether the differences in practice impacted patients’ outcomes. In addition, there is big heterogeneity among Latin-American countries, regions, and institutions. It is difficult to assume that one survey can precisely represent the whole continent’s reality, even though we estimate ~15% of Latin-American centers participated in the latest inquiry. Nevertheless, the results give us a notion of which direction we are moving to and the gaps that still need to be filled, in addition to serving as a guide for the less experienced centers in defining their protocols. Finally, since the WRITTEN survey was not reconducted in the rest of the world during 2019-2020, a direct comparison of the current TAVR practice in Latin America with other centers through the survey’s responses was not possible.

## Conclusion

In conclusion, differences in TAVR practice exist between the Latin America and other developed nations of the world, with an at least 5-year delay in the widespread adoption of some techniques in Latin America. Some of these differences in practice seem to be linked to a lower procedural volume in Latin-American centers, while others could be merely associated with a lack of global consensus and regional variability. Nevertheless, the gap appears to be diminishing since this volume-practice relationship has softened in the latest years due to practice development and the adoption of more refined techniques even by lower volume centers in Latin America. Future studies in the continent are warranted to evaluate the impact of such changes in practice on patients’ clinical outcomes.

## *Supplemental Materials

For additional information, please click here.
